# Powdered Hierarchically Porous Silica Monoliths for the Selective Extraction of Scandium

**DOI:** 10.1021/acssuschemeng.3c04672

**Published:** 2023-10-11

**Authors:** Aaron Brewer, Chloé Reicher, Olivia Manatschal, Hongzhi Bai, Kazuki Nakanishi, Freddy Kleitz

**Affiliations:** † Department of Functional Materials and Catalysis, Faculty of Chemistry, University of Vienna, 1090 Vienna, Austria; ‡ DPS Inc., 615-8530 Kyoto, Japan; § Institute of Materials and Systems for Sustainability, Nagoya University, 464-8601 Nagoya, Japan; ∥ Institute for Integrated Cell-Material Sciences, Kyoto University, 606-8501 Kyoto, Japan

**Keywords:** solid-phase extraction, critical materials, scandium, hierarchically porous monoliths, silica

## Abstract

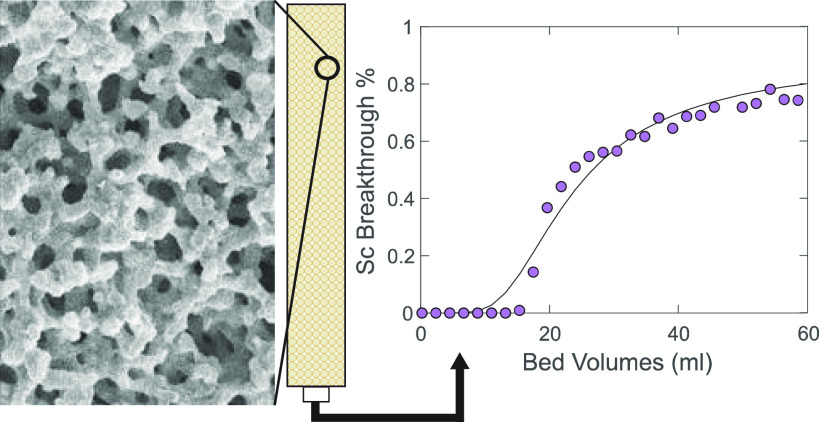

Scandium (Sc) is a high value Critical Material that is most commonly used in advanced alloys. Due to current and potential supply limitations, there has been an international effort to find new and improved ways to extract Sc from existing and novel resources. Solid-phase extraction (SPE) is one promising approach for Sc recovery, particularly for use with low-grade feedstocks. Here, unfunctionalized, powdered hierarchically porous silica monoliths from DPS Inc. (DPS) are used for Sc extraction in batch and semicontinuous flow systems at model conditions. The sorbent exhibits excellent mass transfer properties, much like the whole monoliths, which should permit Sc to be rapidly recovered from large volumes of feedstock. The Sc adsorption capacity of the material is ∼142.7 mg/g at pH 6, dropping to ∼12.0 mg/g at pH 3, and adsorption is furthermore highly selective for Sc compared with the other rare earth elements (REEs). Under semicontinuous flow conditions, recovery efficiency is limited by a kinetic process. The primary mechanism responsible for the system’s slow approach to equilibrium is the Sc adsorption reaction kinetics rather than inter- or intraparticle diffusion. Overall, this unmodified hierarchically porous silica powder from DPS shows great promise for the selective extraction of Sc from various feedstocks.

## Abstract 2

Powder derived from hierarchically porous silica monoliths was used to selectively extract scandium from aqueous feedstocks at model conditions.

## Introduction

Scandium (Sc) is commonly classified as one of the 17 rare earth elements (REEs), and is one of the metals considered to be a Critical Material (CM) by governments around the world, particularly in North America and Europe.^1,2^ This Critical Material designation is based on the importance of Sc for the modern economy combined with its concerning potential supply risks. Scandium is used primarily for lightweight alloys, for example in the aerospace industry, and for solid oxide fuel cells, as well as more minor applications in ceramics and electronics.^1,3^ These uses have driven its price up, and Sc is presently the most valuable of the REEs.^3^ Indeed, its value is high enough that the economic potential of some possible future REE resources may be largely determined by the presence of Sc alone.^4^ However, despite the value and practical utility of Sc, meaningful production of this metal is limited to only a few major sources around the world, with 66% coming from China in 2020.^1^ Since these sources have been and continue to be vulnerable to various geopolitical circumstances, there has been a significant push in many regions to identify new potential Sc feedstocks and to develop new and improved techniques for Sc extraction and purification. Among these emerging novel recovery systems, solid-phase extraction (SPE) is one broad class of metal recovery techniques which employs a solid material to selectively recover a specific component of an aqueous feedstock (i.e., a mineral leachate), generally through adsorption. The purified aqueous metal is then typically precipitated and roasted to produce a salable solid product, such as a metal oxide. SPE can offer advantages, particularly in the area of environmental impact, as these systems typically require limited or no hazardous organic solvents for synthesis or use, unlike traditional liquid–liquid extraction approaches. They are also highly reusable and can function well with low-grade feedstocks like recycled materials or mining wastes,^5^ which represent valuable potential new resources for this metal.^6^ Numerous studies have examined a wide range of materials for the solid-phase extraction of Sc and other REEs from many different feedstocks.

Monolithic structures with both macropore and mesopore regimes, known as hierarchically porous monoliths, have long been an especially tempting target for use as solid-phase extractants for specific metals, such as Sc. The monoliths have so far been comprised of various different materials, including carbon and titania, although silica monoliths are perhaps the most common, as they offer several key advantages for SPE systems.^7^ Silica is particularly promising for Sc extraction, as silica surfaces are inherently able to selectively adsorb Sc without prior modification.^8,9^ The mechanism of this selective Sc adsorption is not yet fully understood, but it is attributed to the silanol functional groups present on the surface of amorphous silica, and a specific structure or conformations of silanol groups may be required for adsorption to occur.^8^ Optimal Sc extraction occurs at a specific pH range (∼3–5) because, at lower pH, the silica surface becomes positively charged, repulsing the Sc^3+^ ion, while at higher pH, Sc precipitation becomes an issue.^8^ Unmodified silica can adsorb certain other metals, such as thorium,^9^ so this effect is not entirely exclusive to Sc, which must be taken into account for some future feedstocks. The monolithic silica structures, in particular, generally exhibit a high surface area for adsorption, and the silica monoliths specifically display exceptional adaptability, due to the range of options for functionalization and excellent tunability in terms of characteristics like pore size, pore volume, and framework structure.^7,10^ Perhaps most importantly, however, the hierarchically porous monoliths have superior mass transport properties compared to traditional particulate adsorbents.^9,11^ This attribute would, in principle, permit large volumes of a given feedstock to be rapidly processed through the sorbent, greatly elevating the potential scalability of the system and circumventing common issues, such as pressure buildup and column clogging. That significant advantage, combined with the other promising characteristics of hierarchically porous silica monoliths, has generated widespread interest in these sorbents for solid-phase extraction applications.

Silica monoliths with the macropore–mesopore structure were first reported in 1991 by Nakanishi and colleagues.^12^ Significant progress has been made since this original study; however, even 30 years later and despite their advantages, whole silica monoliths have, to our knowledge, not yet been applied for selective metal extraction on anything approaching the industrial scale. The primary obstacle to their use appears to be scalability. To establish a point of reference, if one were to assume a relatively low desired single-batch system output of 100 g of Sc and a relatively high sorbent recovery capacity of 100 mg of Sc/g of monolith, the process would require 1 kg of sorbent, which would be approximately equivalent to one 5 L cylindrical monolith. This size is far larger than the largest silica monoliths that have been reported thus far (∼1.1 L),^13^ and there are key obstacles preventing scale-up to that level. First, larger monoliths are highly prone to cracking during drying due to the surface tension exerted by water evaporating in the mesopores.^7,13,14^ This issue can be mitigated by enlarging the mesopore size, for example, through the inclusion of a hydrothermal posttreatment step in the synthesis procedure;^7,13,14^ however, it remains to be seen whether this solution would be sufficient to prevent breakage of even larger monoliths. There are other, more niche, methods to address the cracking problem, such as drying the monoliths through a coating of paraffin oil,^15^ but none present a clear solution for very large monoliths without significant drawbacks. The second issue is that the monolith’s physical characteristics are quite sensitive to temperature during gelation and aging.^7,13,14^ For monoliths with a large diameter, it is difficult to maintain a uniform temperature across the width of the mold during synthesis, which could result in material heterogeneity within the monolith, which is not ideal for highly controlled metal extraction operations.^13^ A theoretical workaround would be to produce many smaller monoliths to use in parallel for Sc extraction. However, this solution has its own drawbacks, namely monolith containment, which is itself a nontrivial obstacle to monolith scale-up. Each individual monolith, regardless of size, must be strictly contained within some type of external sleeve (e.g., a glass or metal cylinder, resin coating, or heat shrink tubing)^16,17^ to retain the liquid feedstock within the system without leaking. If the containment system is not precisely fitted to the monolith, the recovery process will suffer from wall effects and/or channeling and exhibit reduced extraction efficiency. The costs and risks associated with monolith containment are expected to increase with the use of many small monoliths compared with fewer, larger monoliths. Despite decades of research, these scalability issues continue to prevent the use of hierarchically porous silica monoliths for selective metal recovery on the industrial scale.

Powdered silica monoliths may offer an alternative monolith-based sorbent, largely retaining the advantages of single-piece monoliths while avoiding many of their disadvantages. Essentially, a whole monolith is ground and passed through a series of sieves to achieve a powder with a homogeneous particle size. These powders have the same surface chemistry and macropore–mesopore framework (e.g., high surface area) as the whole monoliths, so they are readily functionalized, and their structure can still be easily tuned during the normal monolith synthesis procedure prior to powdering. Furthermore, since the particles still retain the hierarchically porous framework of the whole monoliths, they are also expected to exhibit improved mass transport properties compared to more traditional particulate silica sorbents. The use of powdered monoliths makes the possibility of monolith cracking a nonissue, given that the monoliths will be broken up into small particles anyway. It also minimizes the impact of any monolith heterogeneity given that the fine powder will be mixed and homogenized during grinding. Adsorbent containment is also much more straightforward since the powder can just be packed into a column of any size and shape like the traditional SPE sorbents that have been used for decades. With all of these expected advantages, powdered silica monoliths present a promising material for the recovery of Sc and other Critical Materials.

In this study, the characteristics and selective Sc extraction performance of powdered silica monoliths were assessed. First, the physical attributes of the material are reported and its mass transport behavior is analyzed. Then, the Sc recovery capabilities of the powder are discussed in the context of a series of batch and semicontinuous flow column extraction tests at various environmental conditions. Throughout, these findings are evaluated in the context of the potential of this SPE sorbent for future large-scale metal recovery operations.

## Materials and Methods

Hierarchically porous silica monolith powders were obtained from DPS Inc. in Kyoto, Japan. For the experiment testing column pressure as a function of flow rate, 106–212 μm diameter particles were used, and for all other experiments, 63–105 μm diameter particles were used. There are many options for tuning the physical parameters of these powders, and the characteristics of the selected powder are listed in Table 1. Mesoporous silica spheres (150 μm diameter; SL12SA5) were obtained from YMC Co., Ltd. Aqueous scandium (10000 ppm) and bromide (1000 ppm) standard solutions were acquired from LabKings and Inorganic Ventures, respectively. MES (2-(*N*-morpholino)ethanesulfonic acid) buffer and HomoPIPES (homopiperazine-1,4-bis(2-ethanesulfonic acid)) buffer were acquired from Sigma-Aldrich, and ammonium oxalate and reagent-grade nitric acid were acquired from Alfa Aesar. For column studies, the powder was packed in empty flash columns from Santai Science. The dimensions of the metal extraction columns were 2.2 cm × 12.6 cm, and the dimensions of the mass transport test columns were 4.6 mm × 100 mm.

**Table 1:** Physiochemical Parameters of the Unmodified DPS Monolith Powder and YMC Silica Spheres Derived from N_2_ Physisorption (−196 °C) and Mercury Porosimetry^a^

	*S*_BET_ (m^2^/g)	*d*_meso_ (nm)	*V*_meso_ (cm^3^/g)	*d*_macro_ (μm)	*V*_macro_ (cm^3^/g)	bulk density (g/cm^3^)
DPS	490	8.2	0.58	1	112.5	0.20
YMC	400	12	n/a	n/a	1.1	0.44

a Source data by DPS Inc. and YMC Co., Ltd., Kyoto, Japan.

To determine the mass transport characteristics of the monolith powders compared to traditional spherical sorbents, isopropanol was passed through the fixed-bed columns at 0–6 mm/s. The pump used was a Shimazu LC-20AD. The pressure was measured at the inlet and outlet of the column, and the difference between them was reported as a pressure drop. The reported values are the median of 120 measurements made with the pressure gauge (SMC PSE560-01). Throughout these tests, the temperature was kept at 25 °C and the humidity was kept at 50%. Scanning electron microscopy (SEM) images of the DPS and YMC sorbents were taken on a JEOL Ltd. JSM-6510 instrument.

Batch experiments for this study included an adsorption capacity test at various feedstock concentrations, a kinetics test, and a REE selectivity test, as well as tests at different pH and temperature conditions. To construct an adsorption isotherm, 10 mg aliquots of the DPS powder were exposed to 14.8 mL of a Sc solution from 0 to 100 ppm. The feedstock solutions were all buffered to pH 4 using a 10 mM HomoPIPES buffer and kept at room temperature. For the pH test, 10 mg aliquots of the DPS powder were exposed to 14.8 mL of Sc solution (100 ppm) at room temperature and the pH was varied from 3 to 6. At pH 3, the feedstock was composed of 1 mM nitric acid and was not buffered. At pH 4 and 5, the feedstock was buffered with 10 mM HomoPIPES buffer, and at pH 6, the feedstock was buffered with 10 mM MES buffer. For the temperature test, 10 mg aliquots of the DPS powder were exposed to 14.8 mL of Sc solution (100 ppm) at pH 4 (10 mM HomoPIPES buffer) and placed in an oven or freezer at 5, 25, 40, 50, or 60 °C. Each of these assays was left for 1 week before being sampled for analysis. For the kinetics assay, 10 mg aliquots of the DPS powder were exposed to 14.8 mL of a Sc solution (20 ppm) at pH 4 (10 mM HomoPIPES buffer). The samples were shaken at room temperature for a set period of time from 10 to 10080 min (7 days) before the experiment was concluded. For the REE selectivity test, 10 mg aliquots of the DPS powder were exposed for 1 week to 14.8 mL of a mixed REE solution containing 5 ppm of each REE (except radioactive Pm) at pH 4 and room temperature. In every case, samples of the liquid phase were diluted in 3% nitric acid, and their Sc or REE contents were analyzed on an Agilent 7800 ICP-MS. All experiments were conducted in triplicate.

For the Sc and Br column tests, 8.0 g of monolith powder (dry weight) was suspended in isopropanol (Sigma-Aldrich) and carefully packed into an empty flash column. Following packing, the sorbent beds were first conditioned with ∼2 L of 0.1 mM nitric acid (pH 4). Then, ∼4.5 L of Sc^3+^ solution (50 ppm of Sc, 0.1 mM nitric acid) or ∼0.6 L of bromide (Br^–^) tracer solution (50 ppm Br, 0.1 mM nitric acid) were passed through the column and collected in 7 mL increments. In both experiments, the fluid flow rate through the column was 0.5 mL/min for every step. Prior to analysis, every sixth sample (for the Sc column) or every second sample (for the Br column) was diluted in 3% nitric acid. The Sc and Br contents of the samples were analyzed on an Agilent 7800 ICP-MS.

The results for the adsorption isotherm, the adsorption kinetics, and the breakthrough columns were fitted with various models using nonlinear least-squares curve fitting in Matlab. The Sc adsorption isotherm data were fitted using the standard Langmuir and Freundlich isotherm models. The Sc adsorption kinetics data were fitted with pseudo-first-order, pseudo-second-order, and Weber and Morris models.^18^ The Sc and Br breakthrough behaviors were fitted using a standard and/or fractal-like Bohart–Adams model.^19^ These models are discussed in more detail in Results and Discussion.

## Results and Discussion

### Mass Transfer Properties

**Figure 1:**
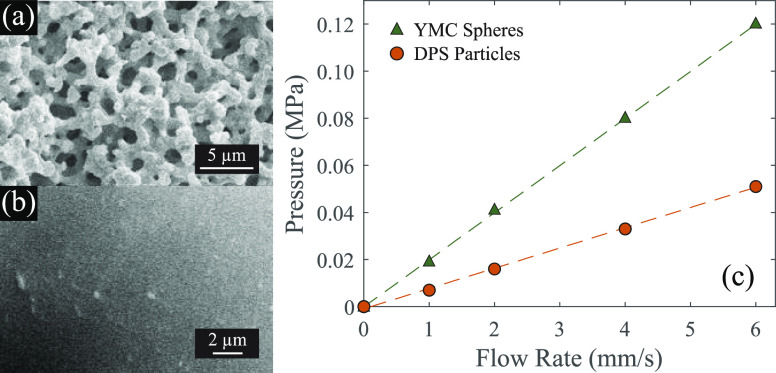
(a) SEM image of a powdered DPS monolith particle exhibiting the characteristic macroporous structure of the material. (b) SEM image of a YMC silica sphere for comparison. (c) Increase in internal column pressure as feedstock flow rate increases for a column packed with the DPS monolith powder vs YMC silica spheres.

The macroporous structure of the hierarchically porous silica monoliths is known to grant these materials excellent mass transport characteristics,^9,13^ and this behavior is expected to be retained in the monolith powders. To test the mass transport characteristics of the powders, the effect of increasing flow rate on internal pressure was observed for two columns, one packed with powdered monolith material (DPS), and the other packed with representative mesoporous silica spheres (YMC). The particles in both columns had a similar size (DPS = 106–212 μm and YMC = 150 μm) and mesopore diameter (DPS = 8.19 nm and YMC = 12 nm). The DPS powder exhibited a typical macroporous structure (1 μm diameter macropores), while the YMC spheres did not. At flow rates between 0 and 6 mm/s, the column pressure was higher for the silica spheres than for the monolith powders, and the pressure difference between the two columns became more exaggerated as the flow rate increased (Figure 1). At a flow rate of 6 mm/s, for example, the pressure of the silica sphere column (0.120 MPa) was more than twice that of the monolith powder column (0.051 MPa; Figure 1). The monolith powders clearly do, at least in large part, retain the advantageous mass transport characteristics of the whole monoliths. This ability to operate at high influent flow rates with substantially decreased column pressure is a significant benefit for industrial-scale metal extraction operations as it would allow large volumes of feedstock to be processed much more rapidly. It is therefore worthwhile to explore the possibilities of using these monolith powders for the selective extraction of critical materials.

### Adsorption Capacity and Selectivity

**Figure 2:**
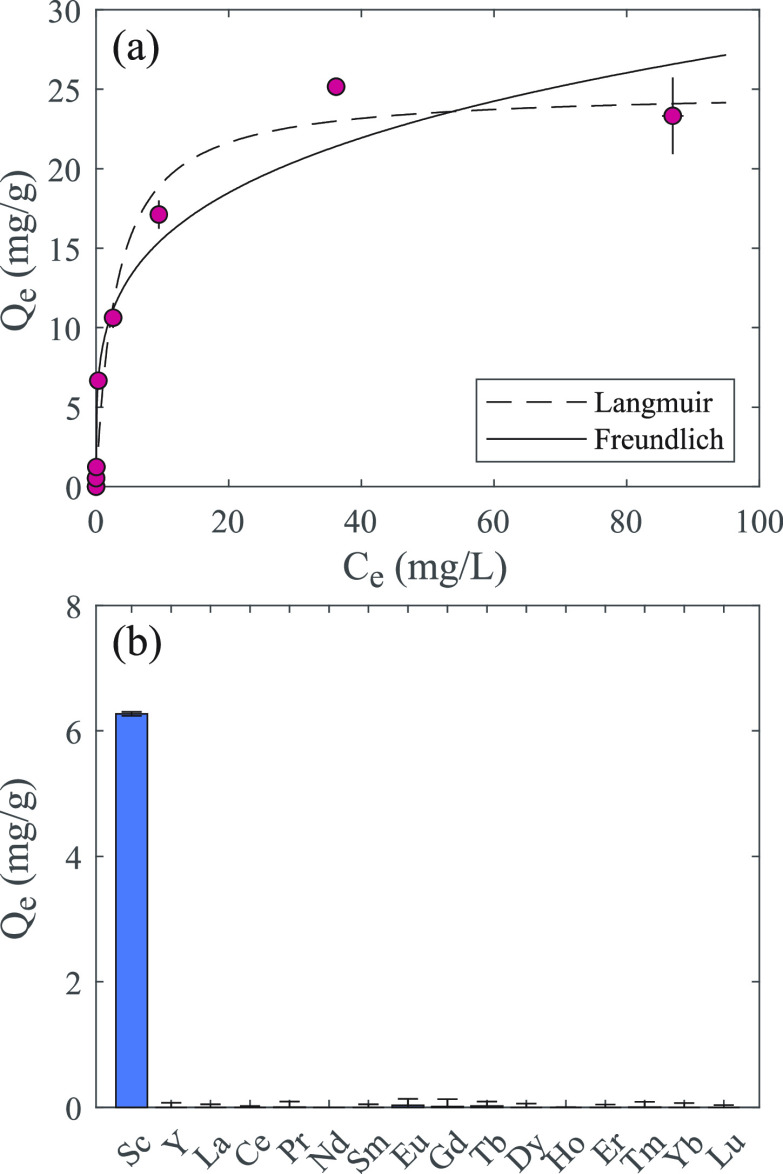
(a) Scandium adsorption isotherm results for the DPS powder fitted with the Langmuir and Freundlich models. *Q*_e_ represents the milligrams of Sc adsorbed per gram of powder. *C*_e_ represents the concentration of Sc remaining in solution following adsorption. The feedstock in each case was kept at pH 4 and 25 °C. (b) REE adsorption results from a solution containing 5 ppm of each individual REE. Error bars represent the standard deviation of triplicates.

**Figure 3:**
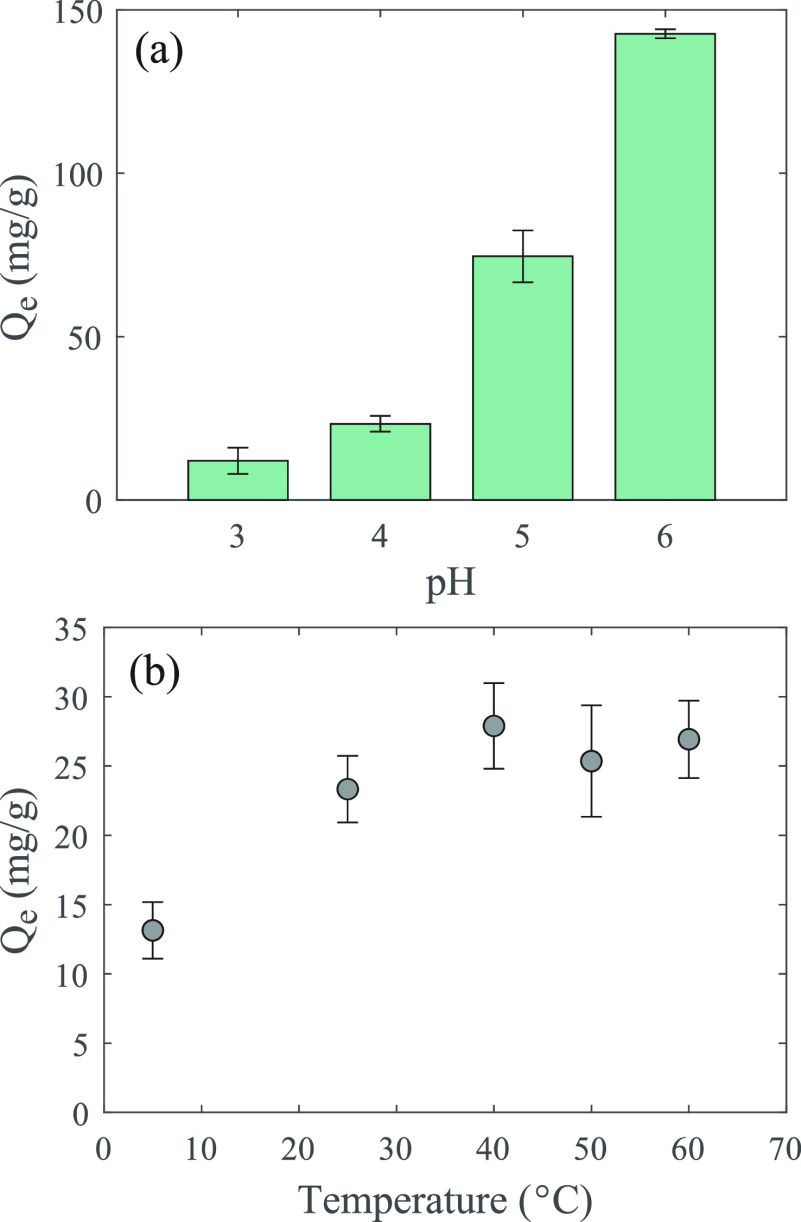
(a) Scandium adsorption results for the DPS powder at pH 3–6. (b) Scandium adsorption results for the DPS powder at 5–60 °C. *Q*_e_ represents the mg of Sc adsorbed per g of powder. Error bars represent the standard deviation of triplicates.

Unmodified silica surfaces are known to have an inherent affinity for Sc adsorption due to the abundance of silanol groups,^8,9,20^ and the silica DPS powder also exhibits this useful property. When exposed to a monoelement Sc solution at pH 4, the powder has a Sc adsorption capacity of ∼24.9 mg/g (Figure 2a). This capacity is well within the range of other published experimental REE sorbents,^21^ and is especially promising given that it was accomplished with only bare silica, without any grafted ligands or other modification. This performance is primarily due to the high surface area of the hierarchically porous powder (490 m^2^/g; Table 1), which results in the exposure of a high number of silanol groups to the aqueous feedstock for a given mass of sorbent.^8,9,20^ The adsorption isotherm (at 25 °C) could be modeled using the typical Langmuir and Freundlich isotherms, with the Langmuir model providing a better fit (*R*^2^ of 0.9631 compared to 0.9468; Figure 2a). The agreement between the Langmuir isotherm and the experimental data suggests that the sorption sites on the silica surfaces are finite and homogeneous, which makes sense given that the sorbent is unmodified silica, and also that sorption is monolayer.^22^ Furthermore, the high adsorption capacity is accompanied by high Sc adsorption selectivity. In an adsorption test in which equal concentrations (∼5 ppm) of each REE were exposed to the DPS powder at pH 4, only Sc was adsorbed (Figure 2b), showing that the powder has a strong preference for Sc adsorption, even compared with the other REEs, which typically exhibit similar behavior to Sc. Adsorption is strongly influenced by pH, with capacity falling to ∼12.0 mg/g at pH 3 and rising to ∼74.6 and ∼142.7 mg/g at pH 5 and 6, respectively (Figure 3a). Temperature plays a smaller role in performance, unlike some other materials,^23,24^ with adsorption capacity appearing to plateau above ∼25 °C, though the capacity is reduced at 5 °C (Figure 3b). These basic Sc adsorption characteristics demonstrate the potential of DPS powder for Sc recovery operations.

In the context of actual industrial-scale Sc extraction, the adsorption behavior of DPS powder presents certain advantages. That strong selectivity for Sc, which is characteristic of unmodified silica, is the first clear benefit. The DPS powder did not adsorb any of the other REEs, and based on the adsorption behavior of other unmodified silica materials, it is not expected to adsorb significant amounts of most abundant non-REE metals.^8,9^ At certain conditions for certain feedstocks, some amount of aluminum and/or thorium may be coadsorbed with the Sc,^8,9^ requiring additional purification steps before or after the SPE step using the DPS powder. At ∼12.0 mg/g at pH 3, the DPS powder also has a high Sc adsorption capacity compared to similar unmodified silica sorbents, such as SBA-15 and KIT-6 at 1.14 mg/g and 1.03 mg/g, respectively.^8^ This performance at pH 3 is important given that the majority of Sc feedstocks are likely to be metal or mineral leachates at very low pH. If the leachate requires less pH adjustment prior to the SPE step, then that is an advantage for the overall extraction system, likely decreasing costs. The material also operates at its peak at a reasonable temperature range for large-scale extraction (25 °C to more than 60 °C), so temperature will typically not be a factor that presents an issue. The performance of the DPS powder in more realistic, semicontinuous flow systems will be assessed in the following sections; however, the fundamental sorption characteristics are promising for practical Sc extraction.

### Fixed-Bed Column Extraction and Adsorption Kinetics

**Figure 4:**
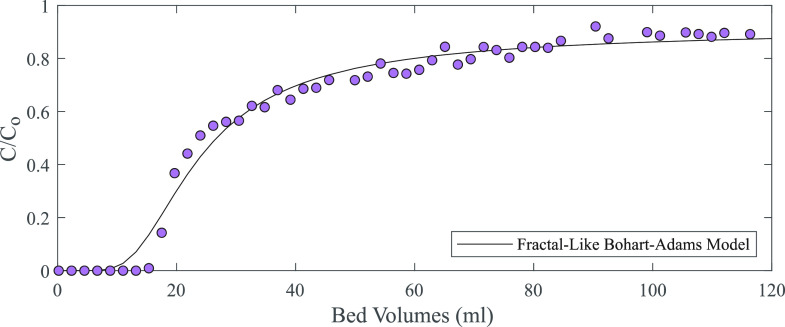
Scandium breakthrough column. The influent was 50 ppm of Sc (mg/L) at pH 4 in 10 mM HomoPIPES buffer. One bed volume for the column was ∼38.8 mL. The solid line is a fractal-like Bohart–Adams model, which is designed to account for intraparticle diffusion limitations.

Larger-scale SPE operations frequently involve continuous or semicontinuous flow of the feedstock through a fixed bed column in order to maximize the volume of feedstock exposed to a given mass of adsorbent and the rate of that exposure, so the DPS powder was tested in a fixed-bed column setup. Eight grams of powder was packed into a 2.2 cm × 12.6 cm column, and ∼4.5 L of 50 ppm of Sc solution (pH 4) was passed through that column at a flow rate of 0.5 mL/min. Scandium breakthrough was observed after ∼17 bed volumes (∼680 mL; Figure 4). The initial breakthrough occurred with a sharp transition, but the approach to equilibrium at 100% breakthrough occurred only gradually, not reflecting the traditional idealized behavior of an extraction column (Figure 4). A total of ∼77.7 mg of Sc (or ∼9.7 mg Sc per g powder) was adsorbed by the extractant over the duration of the experiment. Approximately 38% of that total capacity was reached prior to initial breakthrough, and ∼62% occurred after breakthrough during the gradual approach to equilibrium. The system had not yet reached equilibrium at 100% breakthrough by the conclusion of this test, so strictly speaking, the full adsorption capacity is higher than ∼9.7 mg/g, and a higher fraction of the adsorption occurs after breakthrough. However, this additional capacity would likely be irrelevant as extraction would be stopped prior to that point in order to avoid wasting feedstock when minimal additional extraction is occurring. A practical extraction scheme would most likely stop the recovery process at ∼16 bed volumes and replace the fixed-bed column with a fresh one, which would minimize Sc loss. The adsorbed Sc would then be eluted from the loaded column in a separate step, and the sorbent could be reused. Such a scheme would maximize the advantages of the system (i.e., rapid mass transport, high initial Sc sorption capacity), while minimizing the effects of the slow sorption reaction kinetics.

Given the extent to which the breakthrough behavior exhibited by this system departs from the expected, theoretical behavior, it is clear that Sc recovery was limited by one or more kinetic process. The rate of Sc adsorption onto the silica surfaces could be limited by (1) external diffusion, which in this system would include Sc ion diffusion in the spaces between particles and diffusion within the particle macropores (1 μm in diameter), (2) internal diffusion occurring within the particle mesopores (<10 nm in diameter), and (3) Sc adsorption reaction kinetics on the silanol functional groups.^18^ External diffusion limits are unlikely to be the main cause of the observed behavior, given that the majority of the particle surface area and therefore functional groups for adsorption are expected to be contained within the mesopore structure. Internal diffusion limitations, however, are known to produce similarly asymmetrical breakthrough patterns in other systems.^25^ To investigate this possibility, the breakthrough data was modeled using a fractal-like Bohart–Adams model (eq 1), which has been modified from the traditional Bohart–Adams model specifically in an effort to account for internal diffusion limitations within the sorbent.^19^
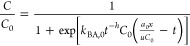
1Here, *C* is the Sc concentration of the effluent, *C*_0_ is the Sc concentration of the influent, *k*_BA,0_ is the fractal-like Bohart–Adams rate constant, *t* is time, *h* is the fractal-like exponent, *a*_0_ is the final adsorption capacity, *x* is the bed height, and *u* is the linear flow velocity. The traditional Bohart–Adams model was unable to be fitted to these breakthrough results at all (data not shown). Even the fractal-like model that is well-suited to account for intraparticle diffusion effects could not provide a precise fit to this highly asymmetrical breakthrough pattern (Figure 4). Interestingly, bromide (Br^–^), a tracer ion that is not adsorbed by the silica, exhibited close to ideal breakthrough behavior (Supporting Information, Figure S1), suggesting that diffusion of the Br^−^ ions through the available pore volumes did not strongly limit the transport of Br through the system. Taken together, these modeling results appear to show that internal diffusion may not be the primary limiting factor in this system.

**Figure 5:**
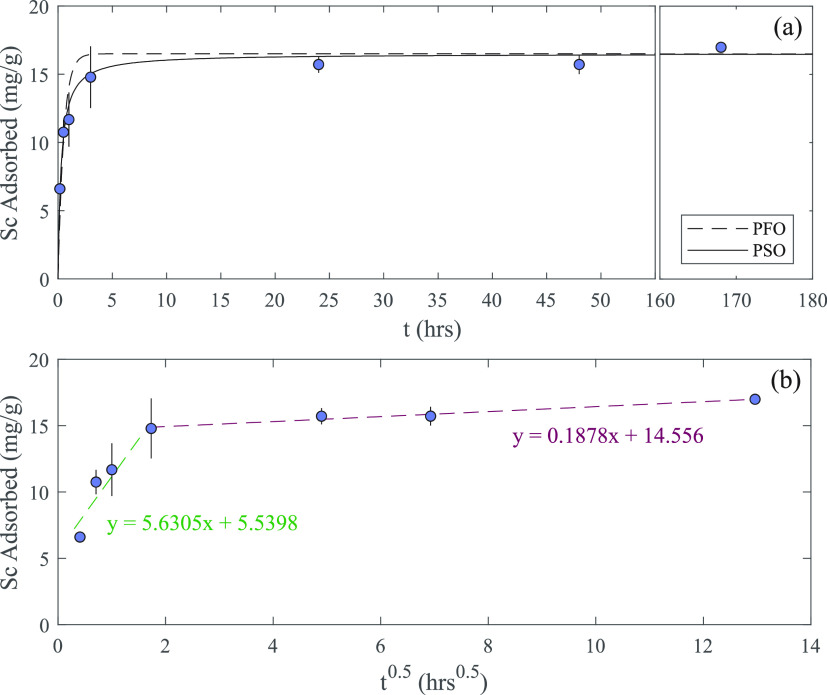
(a) Scandium adsorption kinetics of the DPS powder. The dashed and solid lines represent pseudo-first-order and pseudo-second order kinetics models, respectively. (b) Weber and Morris kinetics model for the same kinetics data. Error bars represent the standard deviation of triplicates.

To further explore the breakthrough behavior exhibited by the DPS sorbent, the Sc adsorption kinetics of the material were analyzed in batch. Aliquots of the DPS powder (10 mg) were gently shaken in ∼15 mL of 20 ppm of Sc solution (pH 4) for a set period of time from 10 min to 7 days (Figure 5a,b). The system reached equilibrium after ∼3 h, following a rapid initial increase in adsorption. Both a pseudo-first-order and a pseudo-second-order kinetics model were applied to the data (Figure 5a).^18^ The pseudo-second-order model provided a better fit (*R*^2^ of 0.9653 compared to 0.8195), which suggests that the adsorption reaction kinetics are likely the limiting factor rather than intraparticle diffusion (Figure 5a).^18,26^ A Weber and Morris model was also applied to the data (Figure 5b), and the intercept of the initial rapid phase of adsorption is well above zero, which again indicates that intraparticle diffusion is not the sole factor influencing extraction kinetics.^18,26^ The batch reaction data and the three models applied to it, in combination with the results of the fractal-like Bohart–Adams model for the continuous-flow system, show that the adsorption reaction kinetics rather than internal diffusion is most likely the primary process controlling the rate of Sc extraction, although small effects from internal and external diffusion may also be present. The mechanism(s) of Sc adsorption onto the silica surfaces are complex, potentially involving van der Waals forces, electrostatic interactions between Sc^3+^ cations and the negatively charged silica, and the formation of covalent Si–O–Sc bonds.^8,27^ While the former two processes occur rapidly, the Si–O–Sc covalent bonds form more slowly,^8,27^ which could explain the observed behavior of the DPS powder in column and in batch. Overall, Sc extraction from the fixed-bed column is limited to an extent by one or more rate-limiting processes within the system, and the slow Sc adsorption reaction kinetics appears to be primarily responsible for this limitation.

The DPS powder did function as a fixed-bed sorbent for Sc recovery under semicontinuous flow, though the breakthrough behavior may present certain difficulties for real-world SPE operations. The equilibrium adsorption capacity at idealized conditions is quite high (∼142.7 mg/g at pH 6), and the selectivity against other REEs is promising. However, if during extraction, the operation must be halted at the breakthrough point, then a large fraction (>68%) of the total adsorption capacity is not used, reducing the practical capacity of the column. If the operation is halted at a later point to take advantage of this additional capacity, significant Sc will have escaped through the column as waste, reducing the extraction efficiency of the system. That being said, the adsorption capacity up to breakthrough (∼3.7 mg/g at pH 4) may still be adequate for extraction operations in some scenarios, especially given the other advantages of this simple system. Typically, highly engineered sorbents such as the DPS powder are functionalized with ligands designed to target specific metals. Eliminating this modification step decreases costs, minimizes the use of potentially hazardous solvents during production, and likely increases material reusability since there is no ligand that can be leached out of the sorbent over time. These promising characteristics may in some cases outweigh the reduced practical adsorption capacity due to the slow reaction kinetics.

Looking into the future, the basic physicochemical properties of the DPS powder provide a solid foundation to build upon for a highly adaptable CM sorbent. In the case of Sc, the DPS powder is expected to function well with feedstocks such as red mud, bauxite residue from aluminum mining that is enriched in Sc, and manufacturing waste from the production of Sc-bearing alloys and fuel cells, just to name two examples. Additional forthcoming testing will assess the extraction performance at more realistic conditions that more accurately reflect the conditions found in leachates from these real-world feedstocks. Further testing to elucidate the nature of the interactions between silanol groups and Sc would also be useful in optimizing the Sc recovery process. To target other desirable metals beyond Sc, the DPS powder can, of course, be functionalized with a wide range of targeted ligands, which could both increase adsorption capacity for a given metal and completely circumvent the kinetics issue by changing the species relevant for the adsorption reactions, though at an increased cost. Other REEs, noble metals, and various other Critical Materials could potentially be extracted and purified with this approach. With or without functionalization, the DPS powder does show great promise for future CM extraction applications.

## Conclusions

The performance of DPS powder as an extractant for batch and semicontinuous flow recovery of scandium was analyzed at conditions relevant for future real-world metal recovery operations. The sorbent exhibited the following characteristics:

1. Mass transfer of fluid feedstocks through a fixed-bed column could occur rapidly without producing significant pressure increases, unlike many powdered SPE sorbents, due to the hierarchical pore structure of the DPS powder.
2. The material, even without surface modification, achieved a high Sc adsorption capacity, ∼24.9 and ∼142.7 mg/g at pH 4 and 6, respectively. Temperature increases above 25 °C had minimal effect on Sc adsorption.
3. The unmodified silica surfaces had a high selectivity for Sc adsorption compared to the other REEs.
4. The DPS powder did function as an extractant for Sc in a semicontinuous flow column system, though optimal performance was reduced by the slow Sc adsorption reaction kinetics, with a potential minor contribution from intraparticle diffusion within the mesopores.
5. The high Sc adsorption capacity and promising selectivity, especially given the lack of surface modification, combined with the excellent mass transfer properties demonstrate that the DPS powder has great potential for the SPE of Critical Materials.

## Supporting Information Available

Tracer ion (Br^–^) breakthrough behavior in the fixed-bed powdered monolith column (PDF)
