# Supplementary material for: Powdered Hierarchically Porous Silica Monoliths for the Selective Extraction of Scandium
Source: ACS Sustain Chem Eng. 2023 Oct 11;11(42):15432–9. doi: 10.1021/acssuschemeng.3c04672 (PMC10598872; doi:10.1021/acssuschemeng.3c04672)
Supplement: Supplementary file 1 — sc3c04672_si_001.pdf [file sc3c04672_si_001.pdf]

Supporting Information

Powdered hierarchically-porous silica monoliths for the selective extraction of scandium

Aaron Brewer,[a*] Chloé Reicher,[a, #] Olivia Manatschal,[a, #] Hongzhi Bai,[b] Kazuki Nakanishi,[c, d] Freddy Kleitz[a*]

[a] Department of Functional Materials and Catalysis, Faculty of Chemistry, University of Vienna, 1090 Vienna, Austria

[b] DPS Inc., 615-8530 Kyoto, Japan

[c] Institute of Materials and Systems for Sustainability, Nagoya University, 464-8601 Nagoya, Japan

[d] Institute for Integrated Cell-Material Sciences, Kyoto University, 606-8501 Kyoto, Japan

Pages: 2

Figures: 1

Tables: 0

*Corresponding authors: Dr. Aaron Brewer and Prof. Dr. Freddy Kleitz

E-mail: aaronbrewer5@gmail.com; freddy.kleitz@univie.ac.at

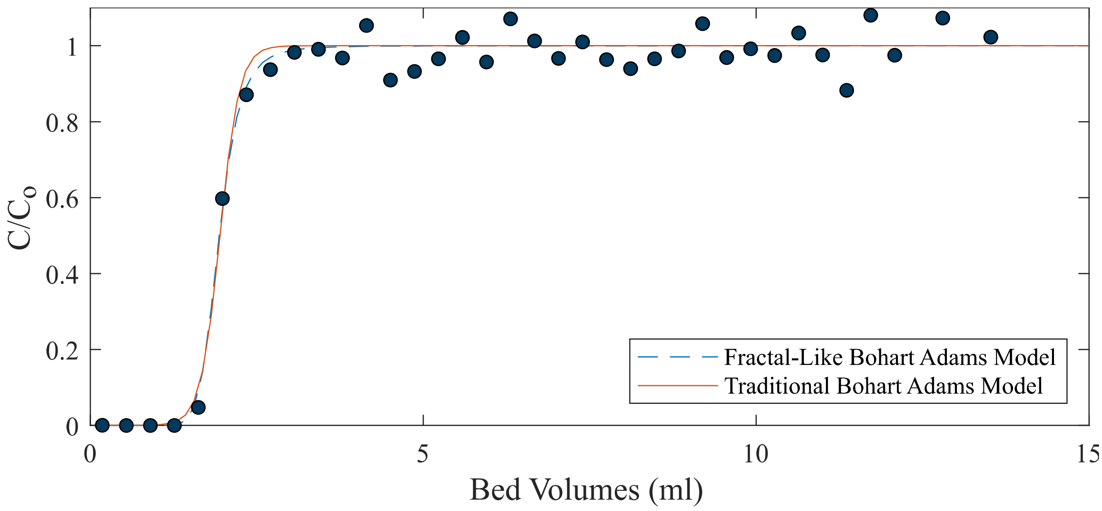

Figure S1. Bromine breakthrough column using the DPS powder. The influent was 50 ppm (mg/L) Br at pH 4 in 10 mM HomoPIPES buffer. One bed volume for the column was ~38.8 ml. The solid red line is a traditional Bohart-Adams model. The dashed blue line is a fractal-like Bohart-Adams model, which is designed to account for intra-particle diffusion limitations. Note that there is minimal difference between them in this system, unlike in the Sc recovery system.
